# Two Hybrid Au-ZnO Heterostructures with Different Hierarchical Structures: Towards Highly Efficient Photocatalysts

**DOI:** 10.1038/s41598-019-53212-3

**Published:** 2019-11-14

**Authors:** Shuo Yang, Lijing Wang, Yongsheng Yan, Lili Yang, Xin Li, Ziyang Lu, Hongju Zhai, Donglai Han, Pengwei Huo

**Affiliations:** 1 School of Chemistry & Chemical Engineering, Jiangsu University, Zhenjiang, 212013 P.R. China; 2 Henan engineering center of new energy battery materials, Henan D&A engineering center of advanced battery materials, College of Chemistry and Chemical Engineering, Shangqiu Normal University, Shangqiu, 476000 P.R. China; 3 Changchun Institute of Optics, Fine Mechanics and Physics, Chinese Academy of Sciences, Changchun, 130033 P.R. China; 4 School of Materials Science and Engineering, Changchun University of Science and Technology, Changchun, 130022 P.R. China; 5 Key Laboratory of Preparation and Applications of Environmentally Friendly Materials of the Ministry of Education, Jilin Normal University, Siping, 136000 Jilin Province P.R. China

**Keywords:** Two-dimensional materials, Photocatalysis

## Abstract

A new paradigm for photocatalysts based on two different hierarchically structured honeycomb and porous cylindrical Au-ZnO heterostructures was successfully developed via a straightforward and cost-effective hydrothermal method under different preparation conditions, which can be promising for industrial applications. The photocatalytic performance of all as-prepared samples under the illumination of sunlight was evaluated by the photocatalytic degradation of rhodamine B (RhB) and malachite green (MG) aqueous solutions. The results show that the photocatalytic degradation efficiency of RhB and MG was 55.3% and 40.7% for ZnO, 95.3% and 93.4% for the porous cylindrical Au-ZnO heterostructure, and 98.6% and 99.5% for the honeycomb Au-ZnO heterostructure, respectively. Compared with those from the ZnO, the results herein demonstrate an excellent reduction in the photoluminescence and improvement in the photocatalysis for the Au-ZnO hybrids with different morphologies. These results were attributed not only to the greatly improved sunlight utilization efficiency due to the surface plasmon resonance (SPR) absorption of Au nanoparticles in the visible region coupled with the UV light utilization by the ZnO nanostructures and multi-reflections of the incident light in the pore structures of their interior cavities but also to the high charge separation efficiency and low Schottky barrier generated by the combination of Au nanoparticles and ZnO micromaterials. Moreover, the honeycomb Au-ZnO heterostructure had a high Au content, surface area and surface oxygen vacancy (O_V_), which enabled photocatalytic properties that were higher than those of the porous cylindrical Au-ZnO heterostructures. In addition, two different formation mechanisms for the morphology and possible photocatalytic mechanisms are proposed in this paper.

## Introduction

Sunlight-driven semiconductor-based heterogeneous photocatalysts have been extensively investigated due to their straightforward operation, environmental friendliness and high efficiency^1–3^. Thus, different semiconductors have been explored to improve their cost-efficiency, long-term stability and efficiency^4–6^. Among these, ZnO stands out due to its excellent characteristics, e.g., a direct wide band gap (Eg = 3.37 eV), good availability, cost effectiveness, low-toxicity, unique acoustic and optoelectronic properties, chemical stability and nanoscale tunability^7–12^. Nevertheless, the wide bandgap energy of ZnO allows the generation of excitons only upon UV light irradiation, which is less than 5% of the solar spectrum, hence limiting its performance as a solar photocatalyst. The relatively high recombination rate of photogenerated electron-hole pairs also greatly reduces the efficiency of ZnO photocatalysts^13–15^.

To overcome the aforementioned drawbacks, a workable solution to improve the optical absorption capacity and reduce charge recombination of ZnO is desired. It is well known that noble metals have desirable properties, such as surface plasmon resonance, straightforward reduction, chemical stability and bio-affinity^16–20^. Among them, Au nanoparticles show excellent SPR properties and charge storage capability^21^. When ZnO is combined with Au nanoparticles, the Schottky barrier at the Au-ZnO interface can effectively separate the photogenerated charge^4,15,22,23^. In other words, a ZnO semiconductor combined with plasmonic Au nanoparticles can substantially enhance the photocatalytic degradation efficiency of organic pollutants^17,24,25^. The performance of Au-ZnO heterostructures largely depends on their preparation method, particle size and morphology^26^. However, the current synthesis methods of Au-ZnO catalysts, including deposition precipitation^27^, co-precipitation^28^, microwave-assisted chemical synthesis^29^, one-pot non-aqueous synthesis^30^ and photodeposition^31^, fail to meet all the needs of cost-efficiency and easy-operation. Moreover, the reported morphologies of Au-ZnO catalysts, including nanorods^32^, nanopyramids^33^, petal-like structures, urchin-like nanoflowers, nanomultipods, nanopyramids^30^ and hollow doughnut-like Au-ZnO catalysts^29^, are too complicated for ordinary laboratory research and industrial applications. To address this issue, we proposed a straightforward, cost-effective and uncomplicated hydrothermal method to synthesize Au-ZnO heterostructures. To further investigate the influence of Au-ZnO structure morphologies on the growth scheme, photoluminescence and photocatalytic performance, honeycomb and porous cylindrical-like Au-ZnO heterostructures were fabricated by adjusting the pH. The new Au-ZnO heterostructures not only were uncomplicated, inexpensive and easy to synthesize but also showed decreased PL intensity and enhanced degradation efficiency, which can be promising for cost-efficient and uncomplicated ordinary laboratory research and industrial applications.

## Experimental Methods

### Materials

All precursors, including chloroauric acid (HAuCl_4_), sodium borohydride (NaBH_4_), ethylene glycol (EG), zinc acetate dihydrate (Zn(CH_3_COO)_2_·2H_2_O), hexamethylene tetramine (C_6_H_12_N_4_, HMT), sodium hydroxide (NaOH) and rhodamine B(RhB), were analytical grade and purchased from Guangdong Chemical Reagent Co. without further purification.

### Preparation of the honeycomb-like Au-ZnO heterostructure

First, Au particles were prepared by dispersing 0.01 mmol NaBH_4_ into 0.01 mmol HAuCl_4_ under continuous stirring until the aqueous solution turned wine red and divided into two equal parts. Second, 0.04 mmol Zn(CH_3_COO)_2_·2H_2_O and 0.02 mmol HMT were added into 40 mL EG. After half an hour, the solution fully reacted and formed white curds; it was then mixed with the Au particles under stirring at room temperature for 2 h. Third, the obtained product was transferred into a Teflon-lined autoclave (18 mL) and heated in an oven at 100 °C for 8 h. After that, the autoclave was cooled naturally to room temperature, and then the sample was repeatedly washed with ethanol to remove any ionic residuals. After drying the sample at 80 °C for 24 h, the honeycomb Au-ZnO heterostructure was finally obtained and kept for further characterization.

### Preparation of the porous cylindrical-like Au-ZnO heterostructure

First, by adding 0.04 mmol Zn(CH_3_COO)_2_·2H_2_O and 0.02 mmol NaOH to 40 mL EG, we obtained pure ZnO after heating the solution to 100 °C for 8 h. Then, part of pure ZnO was kept for future characterization, and the rest was used to prepare the honeycomb Au-ZnO heterostructure. The remaining steps were the same as those for preparing the honeycomb Au-ZnO heterostructures.

### Photocatalytic organic degradation

The photocatalytic activities of the obtained samples were measured by the degradation of the RhB, methyl orange and malachite green aqueous solutions under simulated sunlight. A 500 W Xe lamp with a maximum intensity of 494 nm was used. The pure ZnO and honeycomb and porous cylindrical-like Au-ZnO heterostructures were immersed into the RhB and MG aqueous solution for 30 min in the dark to reach an adsorption-desorption equilibrium between the catalysts and RhB and MG molecules, respectively. In a typical experiment, 0.1 g of the sample was added to 100 mL of 10 mg·L^−1^ organic dye solution. After that, the light source was switched on, and then 2 mL aliquots were withdrawn from every irradiated suspension after 4 min. Then, the concentrations of the RhB and MG were analysed by a UV-Vis spectrophotometer with a distance of 14 cm between the cuvettes and the light source, the absorption spectra were obtained, and the percentage degradation values were calculated.

### Sample characterization

X-ray powder diffraction (XRD) analysis was performed (Rigaku D/max-ga, Japan) at 40 kV and 100 mA and with a Cu radiation source to explore the composition and crystalline properties of the Au-ZnO heterostructures. The scanning speed was 10° min^−1^ from 10° to 80°. Scanning electron microscopy (SEM) images were taken on a LEO-1530VP field-emission scanning electron microscope. High-resolution transmission electron (HRTEM) micrographs and energy dispersive X-ray (EDX) analysis was conducted on a JEOL-2010 high-resolution transmission electron microscope with an accelerating voltage of 220 kV. X-ray photoelectron spectroscopy (XPS) was performed on an ESCALAB 250 (Thermo Scientific, Grand Island, NY, USA) with a monochromatized Al K X-ray source (1486.6 eV) and 500 μm spot size. Photoluminescence (PL) spectra were measured at room temperature at an excitation wavelength of 325 nm, and UV-vis absorption spectra were explored on a TU-1901 spectrophotometer. The BET specific surface areas were measured at −196 °C using an ASAP 2010 analyser (Micromeritics, Norcross, GA, USA).

## Results and Discussion

**Figure 1:**
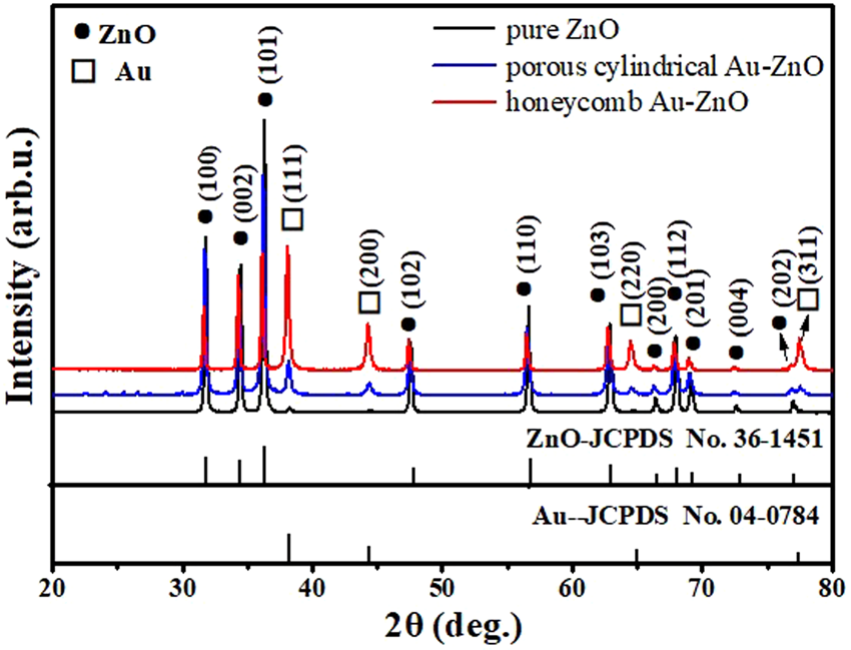
XRD patterns of pure ZnO (black line) and honeycomb (red line) and porous cylindrical (blue line) Au-ZnO heterostructures.

Figure 1 presents the XRD patterns of the as-prepared pure ZnO (black line) and honeycomb (red line) and porous cylindrical (blue line) Au-ZnO heterostructures. As seen for the black line in Fig. 1, all the diffraction peaks observed at 2θ values of 31.7°, 34.4°, 36.2°, 47.5°, 56.6°, 62.9°, 66.3°, 67.9°, 69.1°, 72.6°, and 76.9° matched well to the (100), (002), (101), (102), (110), (103), (200), (112), (201), (004) and (202) planes, respectively, for hexagonal wurtzite ZnO (JCPDS card No. 36-1451)^34^. As the XRD patterns of the honeycomb and porous cylindrical Au-ZnO samples showed, the diffraction peaks (except for that from ZnO) observed at 2θ values of 38.1°, 44.2°, 64.5° and 77.5° were indexed to the (111), (200), (220) and (311) planes, respectively, for cubic phase Au (JCPDS No. 04-0784)^35^. No characteristic peaks of other impurities were detected, indicating that the films were prepared as intended. In addition, the Au peak intensity from the honeycomb Au-ZnO was stronger than that from the porous cylindrical-like Au-ZnO heterostructures, indicating that the Au content in honeycomb-like Au-ZnO heterostructures was higher than that in cylindrical-like Au-ZnO heterostructures.

**Figure 2:**
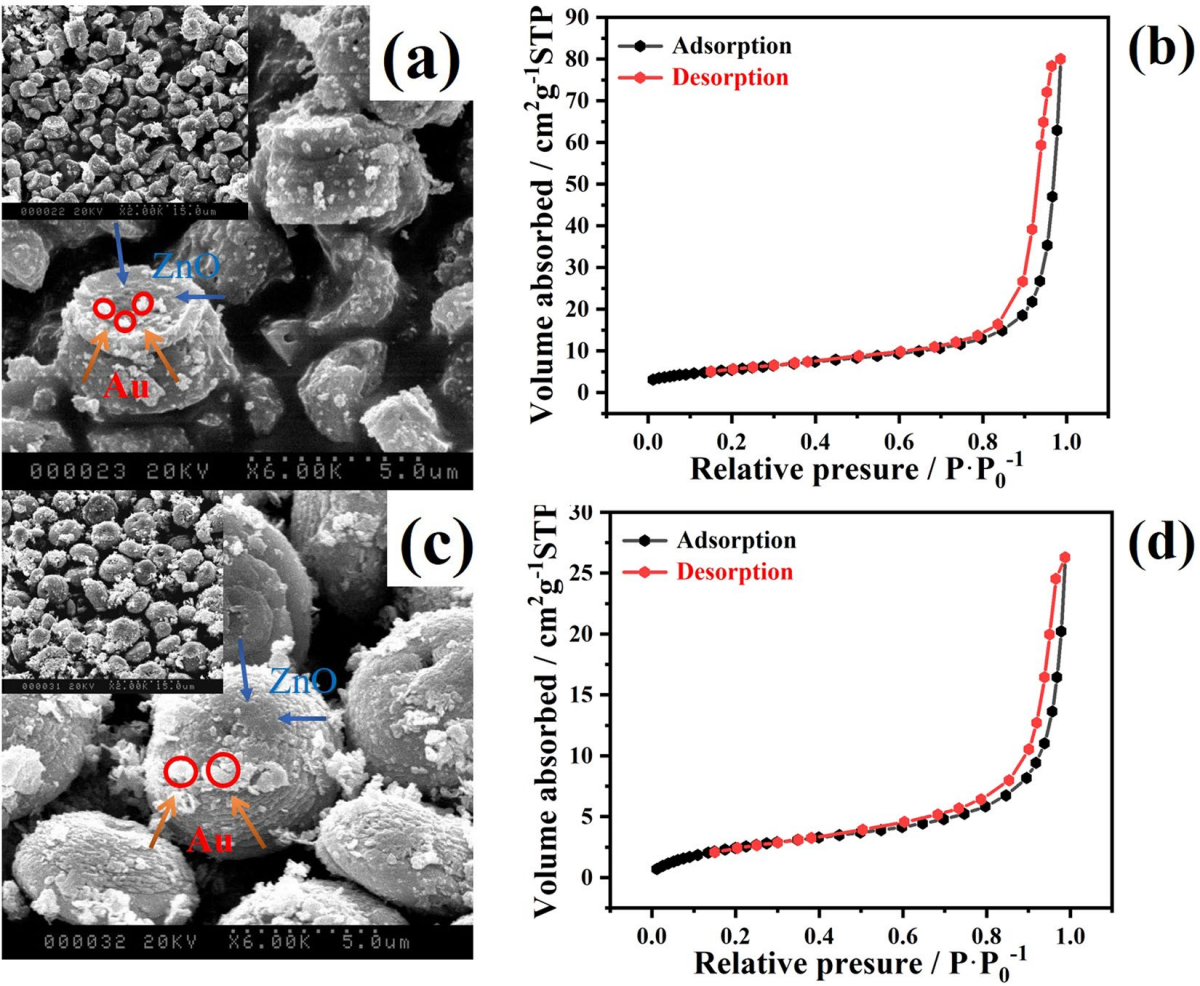
(**a**) SEM image and (**b**) BET plot for the honeycomb Au-ZnO heterostructures, and (**c**) SEM image and (**d**) BET plot for the porous cylindrical Au-ZnO heterostructures.

The morphology evolution was investigated with SEM and BET tests were carried out to observe the amount of Au nanoparticles, morphology and specific surface area of the honeycomb and porous cylindrical Au-ZnO heterostructures. Figure 2(a) shows an SEM image of the honeycomb-like Au-ZnO heterostructure. Au nanoparticles were uniformly distributed across the ZnO surface, and the Au-ZnO heterostructure consisted of many hollow structures with an outer diameter of 5 μm and an inner diameter of approximately 150 nm. Figure 2(c) shows an SEM image of the porous cylindrical-like Au-ZnO heterostructure, which contains a block-like structure with many small holes. The N_2_ adsorption-desorption isotherms of the honeycomb and porous cylindrical Au-ZnO heterostructures are presented in Fig. 2(b,d), respectively. The isotherms of the two samples exhibited the characteristics of type H3 hysteresis loops based on the Brunauer–Deming–Deming–Teller (BDDT) classification for a high relative pressure between 0.4 and 1.0^36^. The analyses show that the BET specific surface area of the honeycomb and porous cylindrical Au-ZnO heterostructures was 20.73 and 10.08 m^2^·g^−1^, respectively. In addition, the average pore size for the honeycomb and porous cylindrical Au-ZnO heterostructures was approximately 23.89 and 16.13 nm, respectively. In comparison to that of the porous cylindrical Au-ZnO, the pore size of the honeycomb Au-ZnO were smaller, which resulted in a high amount of exposed surface area and increased absorption of the solar light. This publication also suggested that pore structures could allow multi-reflections of light radiation in their interior cavities, leading to enhanced light harvesting and photocatalytic activity^37^.

**Figure 3:**
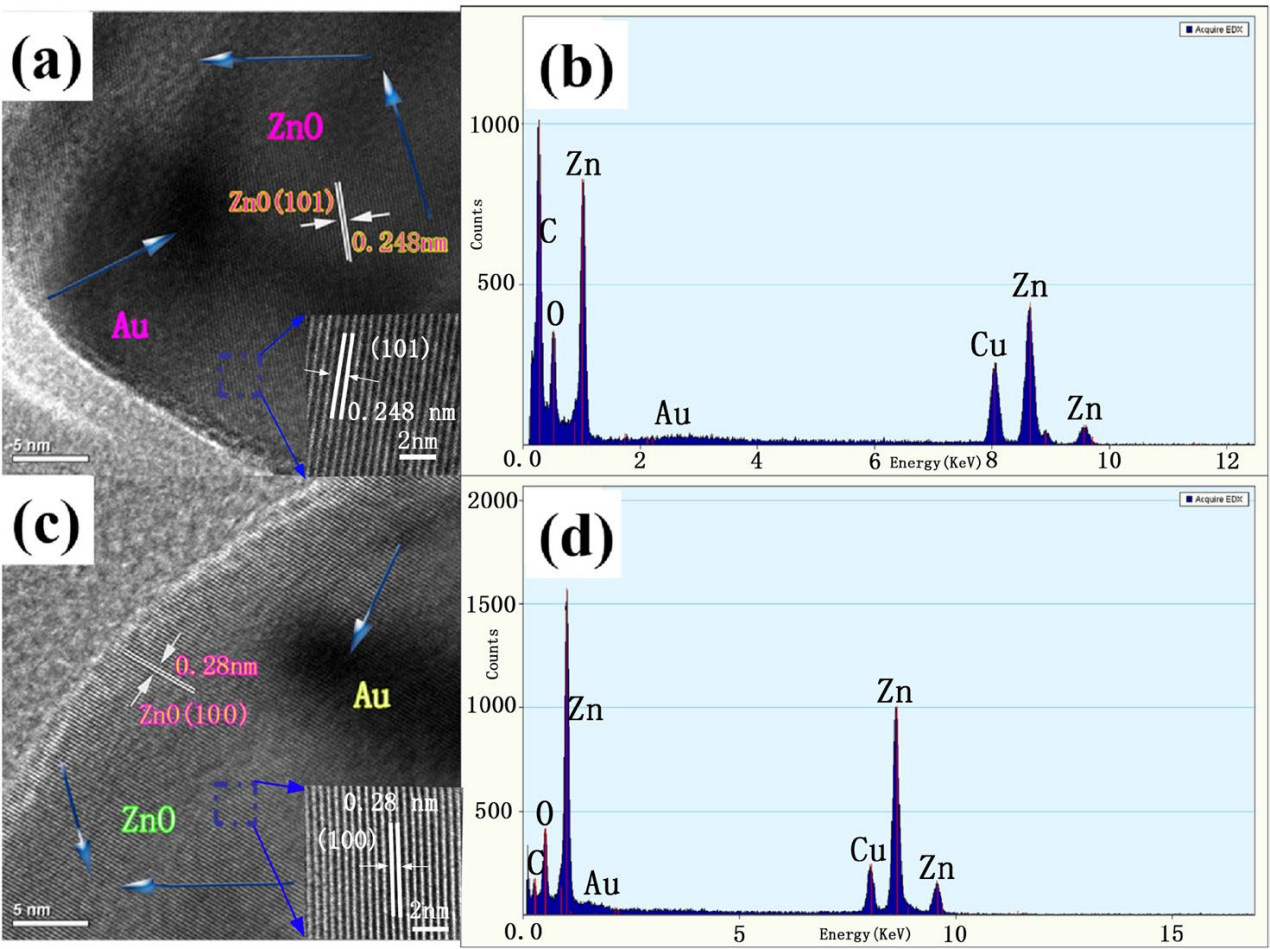
(**a**) HRTEM, magnified HRTEM patterns and (**b**) EDX spectra of honeycomb Au-ZnO heterostructures and (**c**) HRTEM, magnified HRTEM patterns and (**d**) EDX spectra of porous cylindrical Au-ZnO heterostructures.

To further identify the morphology and detailed structure of the Au-ZnO heterostructures and reveal their chemical element composition, HRTEM and EDX analyses of the honeycomb (Fig. 3a,b) and porous cylindrical Au-ZnO heterostructures (Fig. 3c,d) were carried out. As shown in Fig. 3(a,c), the distance between two adjacent planes in wurtzite ZnO was determined to be 0.248 nm (honeycomb) and 0.28 nm (porous cylindrical), corresponding to the (101) and (100) planes of ZnO, respectively. These crystal planes had clear fringes, and the shades between them varied, indicating the formation of the Au-ZnO heterostructures. Additionally, the EDX spectra of the honeycomb (Fig. 3b) and porous cylindrical (Fig. 3d) structures were obtained to determine their chemical composition. The analysis results showed that both structures contained Zn, Au, and O elements, which clearly indicated the formation of Au-ZnO heterostructures. The concentration of Au particles in the honeycomb heterostructure (Fig. 3b) was higher than that of the porous cylindrical Au-ZnO heterostructure (Fig. 3d), which is consistent with the XRD and SEM results.

**Figure 4:**
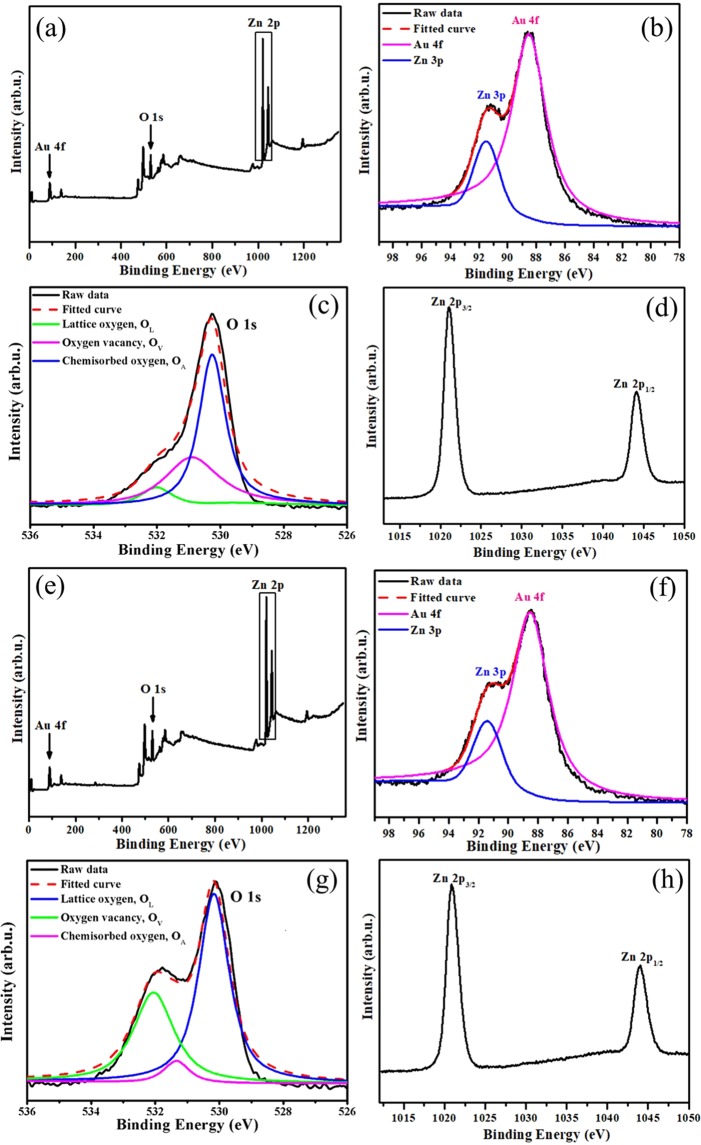
Data for (**a–d**) honeycomb and (**e–h**) porous cylindrical Au-ZnO heterostructures: XPS survey spectra (**a,d**) and high-resolution XPS spectra of Au 4 f (**b,e**), O 1 s (**c,g**) and Zn 2p (**d,h**).

XPS measurements were performed to investigate the surface elemental composition and elemental valences of the honeycomb (Fig. 4a–d) and porous cylindrical Au-ZnO heterostructures (Fig. 4e–h). All binding energy values in the XPS spectra were calibrated according to the information for C 1 s (284.6 eV)^38^. The presence of C mainly originated from the oil pump due to the vacuum treatment^39^. In the survey spectra of the honeycomb and porous cylindrical Au-ZnO heterostructures (Fig. 4a,e, respectively), all elements, namely, Au, O and Zn, were detected with strong characteristic peaks. The O 1 s peaks were fitted into three peaks located at 530.1, 531.0, and 532.0 eV, as shown in Fig. 4c,g, indicating three different kinds of oxygen species in both the honeycomb and porous cylindrical Au-ZnO heterostructures. The oxygen peak at 532.0 eV was ascribed to lattice oxygen (O_L_) in the wurtzite structure that was surrounded by zinc atoms that had their full complement of nearest-neighbour O^2−^ ions. The medium oxygen peak at 531.0 eV was ascribed to O^2−^ ions in oxygen vacancy (O_v_) regions within the ZnO matrix^40^. The oxygen peak at 530.1 eV was attributed to chemisorbed oxygen (O_A_) caused by the surface hydroxyl groups (O-H bonds)^40^. Upon comparing Fig. 4c,g, the O_v_ peak from the honeycomb Au-ZnO was stronger than that from the porous cylindrical Au-ZnO, which is consistent with the result that the photocatalytic performance of the honeycomb Au-ZnO was better than that of the porous cylindrical Au-ZnO^41^. The Zn 2p regions in the XPS spectra (Fig. 4d,h) consisted of two peaks centred at 1021.9 eV and 1044.9 eV, which are characteristic of Zn 2p3/2 and Zn 2p1/2 in ZnO^42^. The Zn 2p core level dipoles induced by spin-orbit coupling are typical of ZnO materials in terms of binding energy, peak shape and peak separation, which was 23 eV^43^.

**Figure 5:**
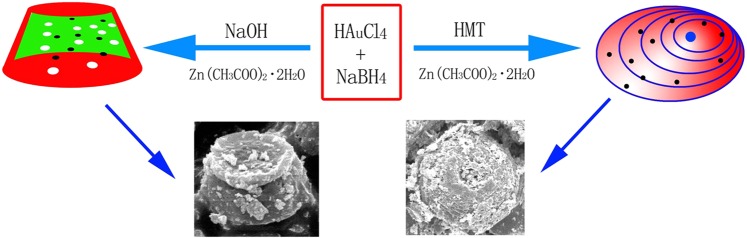
Scheme of the preparation of honeycomb and porous cylindrical Au-ZnO heterostructures.

Based on the experimental results above, the synthesis scheme of the two Au-ZnO heterostructures is presented in Fig. 5, which clearly describes the differences between the two samples during the preparation process. As discussed above, the Au concentration in the honeycomb Au-ZnO heterostructure was higher than that of the porous cylindrical Au-ZnO heterostructure, which was closely related to the use of NaOH. The honeycomb Au-ZnO was prepared with HMT, while the porous cylindrical Au-ZnO was prepared with NaOH. HMT can effectively combine with Zn^2+^ and maintain Zn^2+^ at a low concentration. HMT can also coordinate to the ZnO crystal, obstructing the growth of certain surfaces^44^. According to previous systematic studies, HMT can control the morphology of different samples. Here, HMT acted as a pH buffer by slowly decomposing the NH_3_ and combining with the Zn^2+^ to form [Zn(NH_3_)_4_]^2+^ ^45^, which resulted in the corrosion of the ZnO and the formation of a honeycomb Au-ZnO heterostructure. During the growth of the porous cylindrical Au-ZnO heterostructures, the NaOH reacted with the Zn(CH_3_COO)_2_·2H_2_O to form Zn(OH)_2_ but decomposed to ZnO at high temperatures. However, the strong alkaline hydroxide may partially etch ZnO and cause the formation of [Zn(OH)_4_]^2−^, which helped form the porous structure. It is believed that the porous cylindrical Au-ZnO had a weaker corrosion degree than that of the honeycomb Au-ZnO. Thus, the honeycomb Au-ZnO heterostructure exhibited a higher concentration of Au and a larger surface area than that of the porous cylindrical Au-ZnO. The reaction equations are as follows ((1–4) for honeycomb Au-ZnO, while (1 and 5–7) is for porous cylindrical Au-ZnO):

1 $${{\rm{HAuCl}}}_{4}\,\underrightarrow{{{\rm{NaBH}}}_{4}}\,{\rm{Au}}$$

2 $${({{\rm{CH}}}_{2})}_{6}{{\rm{N}}}_{4}+6{{\rm{H}}}_{2}{\rm{O}}\to 4{{\rm{NH}}}_{3}+6{\rm{HCHO}}$$

3 $${{\rm{Zn}}}^{2+}+2{{\rm{NH}}}_{3}+2{{\rm{H}}}_{2}{\rm{O}}\to {\rm{Zn}}{({\rm{OH}})}_{2}+2{{\rm{NH}}}_{4}^{+}$$

4 $${\mathrm{Zn}(\mathrm{OH})}_{2}+4{{\rm{NH}}}_{3}\to {[{\rm{Zn}}{({{\rm{NH}}}_{3})}_{4}]}^{2+}+2{{\rm{OH}}}^{-}$$

5 $${\rm{NaOH}}+{\mathrm{Zn}(\mathrm{CH}}_{3}{\mathrm{COO})}_{2}\to {\mathrm{Zn}(\mathrm{OH})}_{2}+2{{\rm{CH}}}_{3}{\rm{COONa}}$$

6 $${\mathrm{Zn}(\mathrm{OH})}_{2}+2{{\rm{OH}}}^{-}\to {[{\rm{Zn}}{({\rm{OH}})}_{4}]}^{2-}$$

7 $${\rm{Zn}}{({\rm{OH}})}_{2}\to {\rm{ZnO}}+{{\rm{H}}}_{2}{\rm{O}}$$

**Figure 6:**
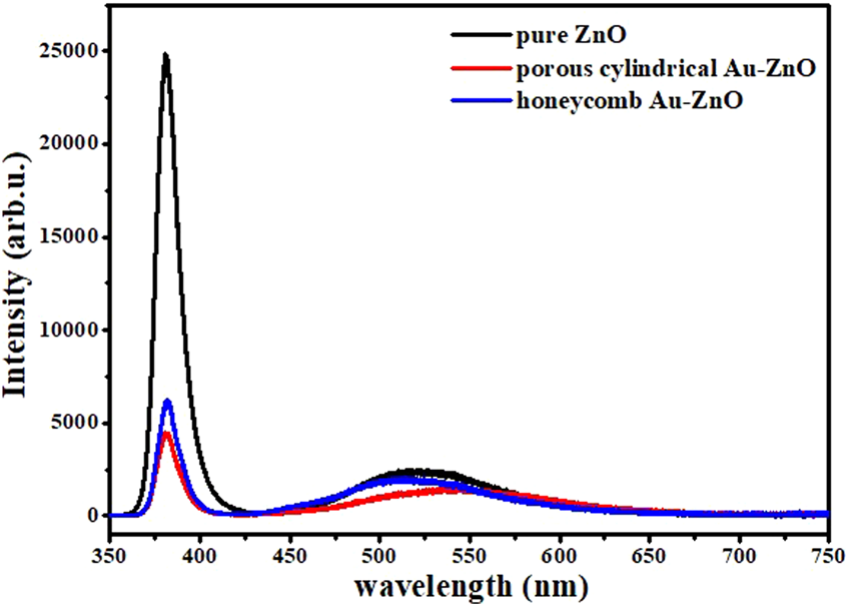
PL spectra of pure ZnO (black line) and honeycomb-like (red line) and porous cylindrical-like (blue line) Au-ZnO heterostructures.

Figure 6 presents the PL spectra for the pure ZnO and Au-ZnO heterostructures with different morphologies. Two ZnO peaks were detected, namely a sharp and narrow peak located at 380 nm and a low and broad peak at approximately 600 nm; no peak was detected from the Au particles. Due to the influence of the Au particles, the peak intensity of Au-ZnO heterostructure showed an obvious decrease in both the ultraviolet and visible light regions. The honeycomb Au-ZnO heterostructure exhibited a lower peak intensity than that of the porous cylindrical Au-ZnO heterostructure, and its peak in the visible region was slightly shifted to the right. Generally, the near-band ultraviolet emission of ZnO at approximately 380 nm is considered an exciton transition, while the emission peak between 520 and 600 nm is considered a deep-level emission caused by oxygen vacancies or Zn vacancy defects^46^. There are two possible reasons for the different emission intensities compared to those in the pure ZnO. First, the PL emission mainly results from the recombination of excited electrons and holes^47^. A decreased PL intensity indicates an increased separation efficiency, and the weak UV and visible emission intensity of the Au-ZnO heterostructure indicates a lower recombination rate for the photoelectron carriers than that for pure ZnO. This is due to the electrons excited from the valence band to the conduction band transfer to the Fermi level of Au, thereby preventing the direct recombination of electrons and holes. Second, it has been reported that PL emission is affected by the number and size of Au nanoparticles^48^. When the number of Au nanoparticles increases or the size of Au nanoparticles surpasses an optimum value, the intensity of the near-band-edge emission is reduced. From the SEM images, we can see that the honeycomb Au-ZnO possessed more Au nanoparticles with a larger mean diameter than those in the porous cylindrical Au-ZnO. The redshift was mainly attributed to the interfacial interaction between the ZnO and Au, which might lead to a charge variation of the Au surface.

**Figure 7:**
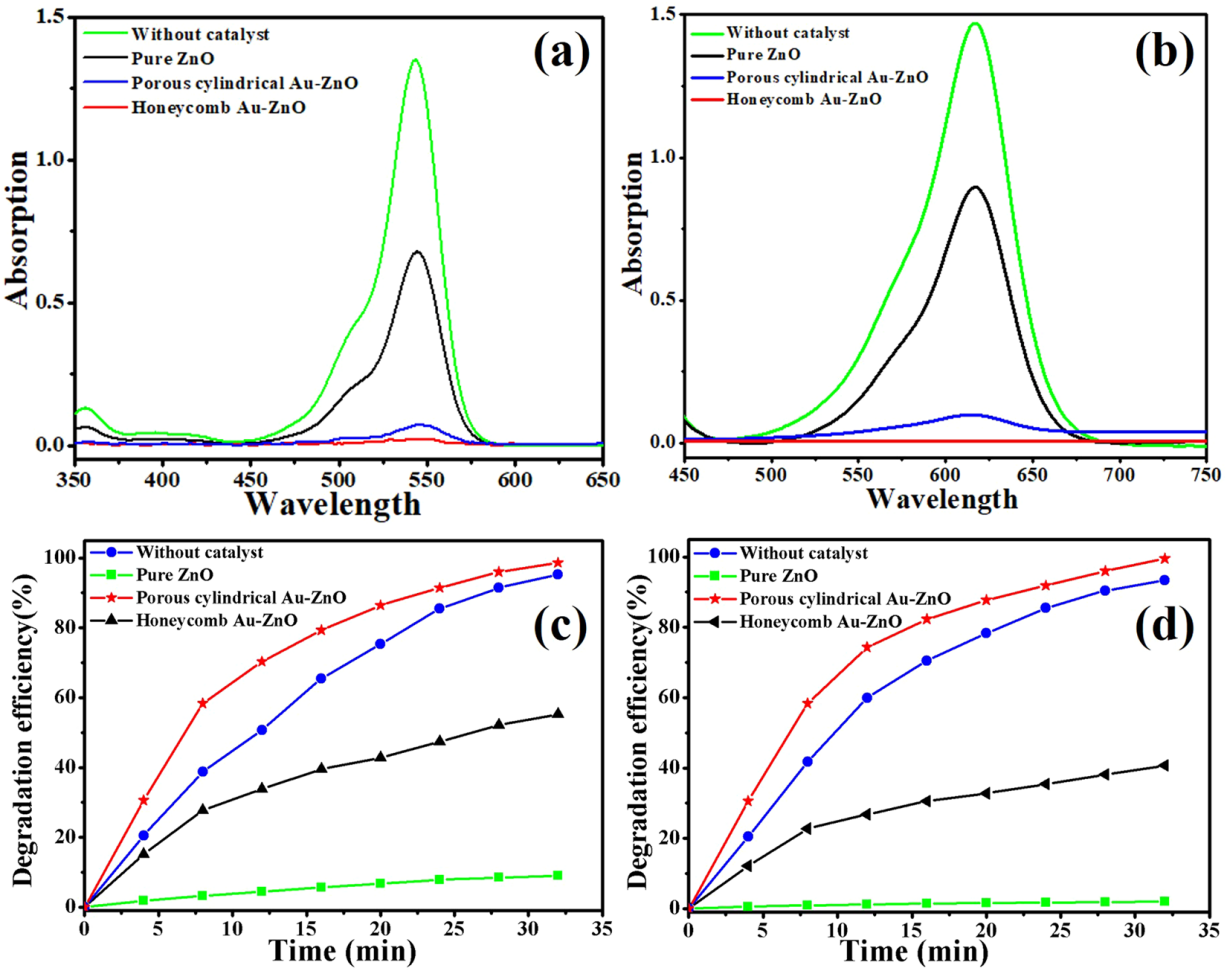
The absorption spectra and degradation efficiency as a function of time under simulated sunlight for the RhB (**a,c**) and MG (**b,d**) solutions without a sample (green line), with pure ZnO (black line) and with the honeycomb (red line) and porous cylindrical (blue line) Au-ZnO heterostructures.

The photocatalytic activities of the pure ZnO and two Au-ZnO heterostructures were investigated by degrading RhB and MG under simulated sunlight, and the corresponding UV-vis absorption spectra are shown in Fig. 7. From Fig. 7(a,b), we can see a strong absorption peak for RhB centred at approximately 550 nm and a strong absorption peak for MG centred at approximately 614 nm. Without a catalyst, it hardly decreased within 32 min, and the pure ZnO showed certain catalytic properties. In comparison, a distinct enhancement in the photocatalytic efficiencies was observed when using the Au-ZnO heterostructure, with a rapid decrease of the main absorption peak intensity. It can be seen that the RhB and MG were almost fully degraded within 32 min. The degradation rates of the RhB and MG are shown in Fig. 7c,d, respectively, which reveals their degradation efficiency clearly by comparing the photocatalytic activities of the three different catalysts: honeycomb (98.6% and 99.5%, respectively), porous cylindrical Au-ZnO heterostructure (95.3% and 93.4%, respectively) and pure ZnO (55.3% and 40.7%, respectively). It is well known that the photocatalytic activity is mainly determined by the phase structure, adsorption ability, and separation efficiency of photogenerated electrons and holes. As Au and ZnO contact each other, electrons in the ZnO migrate to the conduction band of the Au and generate a shortstop Schottky barrier at the interface of the two materials^49^ thus, the Au-ZnO composites exhibited higher photocatalytic activity than that of the pure ZnO. In addition, the honeycomb Au-ZnO heterostructure had a higher Au content, surface area and surface O_V_ than those of the porous cylindrical Au-ZnO heterostructure, which enabled its relatively high catalytic properties.

### Mechanism

**Figure 8:**
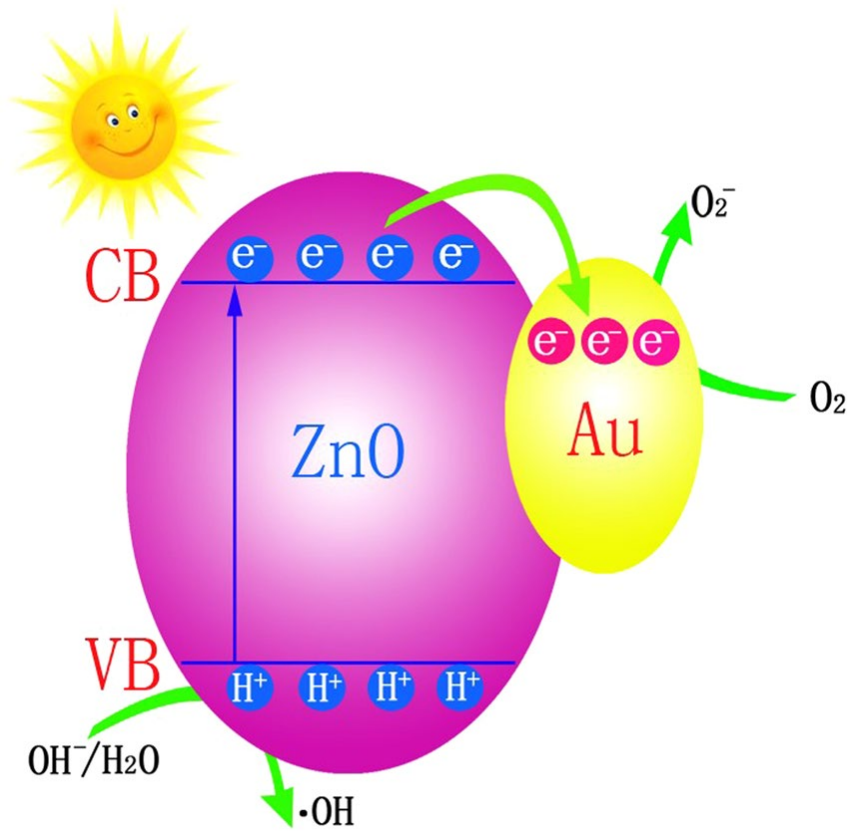
Mechanism for the degradation procedure of RhB with Au-ZnO heterostructure catalysts.

As shown in Fig. 8, a possible mechanism for the Au-ZnO catalyst behaviour is proposed. Under direct sunlight, excited electrons from the ZnO surface are driven to the Au nanoparticles because they have a stronger electron capture capability than that of the ZnO. The excited electrons combine with O_2_ and form •O_2_^**−**^ on the surface of Au nanoparticles^50^. Furthermore, the electron transfer facilitates the formation of holes (H^+^) on the ZnO surface, leading to the production of •OH. Both •O_2_^**−**^ and •OH are active substances for degrading organic dyes, which results in a significant enhancement in the catalytic activity^51,52^. In addition, the unique honeycomb and cylindrical structures with holes contribute to the large surface area, guaranteeing full contact between the light and RhB. Therefore, the catalytic activity is further enhanced. The pore structures also provide the organic dyes and catalysts with pathways in and out, which greatly accelerates the reaction^53,54^. Finally, the honeycomb and porous cylindrical structures allow multiple reflections of solar light, which enhances the light harvesting and increases the distance of the photogenerated electrons and holes, which greatly enhances the catalytic properties of the samples.

## Conclusion

In summary, honeycomb and porous cylindrical Au-ZnO heterostructures were obtained through a straightforward, cost-effective and uncomplicated hydrothermal method. A possible formation and degradation mechanism were proposed. The two new heterostructures created a high surface area and multiple sunlight reactions via special pore structures, which resulted in their decreased PL intensity and enhanced degradation efficiency. Owing to the relatively high Au concentration and surface area, the honeycomb Au-ZnO heterostructures exhibited weaker PL intensities and higher degradation efficiencies than those of the porous cylindrical Au-ZnO heterostructures. With the honeycomb Au-ZnO catalyst, the RhB and MG solutions were almost completely degraded after 32 min. It is expected that the Au-ZnO heterostructures may have various technological applications, such as in photocatalysts, solar energy conversion and environmental purification.
